# Induced cancer stem cells generated by radiochemotherapy and their therapeutic implications

**DOI:** 10.18632/oncotarget.14230

**Published:** 2016-12-26

**Authors:** Xiewan Chen, Rongxia Liao, Dezhi Li, Jianguo Sun

**Affiliations:** ^1^ Medical English Department, College of Basic Medicine, Third Military Medical University, Chongqing, China; ^2^ Cancer Institute of PLA, Xinqiao Hospital, Third Military Medical University, Chongqing, China

**Keywords:** induced cancer stem cells, dedifferentiation, reprogramming, plasticity, therapeutic resistance

## Abstract

Local and distant recurrence of malignant tumors following radio- and/or chemotherapy correlates with poor prognosis of patients. Among the reasons for cancer recurrence, preexisting cancer stem cells (CSCs) are considered the most likely cause due to their properties of self-renewal, pluripotency, plasticity and tumorigenicity. It has been demonstrated that preexisting cancer stem cells derive from normal stem cells and differentiated somatic cells that undergo transformation and dedifferentiation respectively under certain conditions. However, recent studies have revealed that cancer stem cells can also be induced from non-stem cancer cells by radiochemotherapy, constituting the subpopulation of induced cancer stem cells (iCSCs). These findings suggest that radiochemotherapy has the side effect of directly transforming non-stem cancer cells into induced cancer stem cells, possibly contributing to tumor recurrence and metastasis. Therefore, drugs targeting cancer stem cells or preventing dedifferentiation of non-stem cancer cells can be combined with radiochemotherapy to improve its antitumor efficacy. The current review is to investigate the mechanisms by which induced cancer stem cells are generated by radiochemotherapy and hence provide new strategies for cancer treatment.

## INTRODUCTION

Malignant tumors caused over 8 million deaths worldwide in 2013 and are the second leading cause of death behind cardiovascular disease according to the report of Global Burden of Disease (GBD) [1]. Most patients are in the middle and late stages at the first diagnosis, and therefore radiotherapy and/or chemotherapy (abbreviated as radiochemotherapy) becomes their essential choice. However, local and distant tumor relapse generally occurs in patients receiving radiochemotherapy. The mechanisms underlying tumor recurrence and radiochemotherapy resistance remain unclear.

Emerging evidences indicate that preexisting cancer stem cells (CSCs) are responsible for treatment resistance and subsequent progression, recurrence, and metastasis of cancer [2–4]. For the origin of preexisting CSCs, it has been established that normal stem cells and differentiated somatic cells can be transformed into CSCs under certain conditions. However, recent studies reveal that ionizing radiation and chemotherapy can induce reprogramming or dedifferentiation of non-stem cancer cells and generate induced cancer stem cells (iCSCs) [5–9]. These findings imply that radiochemotherapy itself is a possible cause of tumor recurrence and metastasis. To date, no systematic review has been drawn on radiochemotherapy-driven iCSCs. The current review aims to integrate the latest findings on iCSCs and to provide a possibly revolutionary alternative for cancer treatment.

## DEDIFFERENTIATION AND INDUCED CANCER STEM CELLS (ICSCS)

Preexisting CSCs, alternatively called tumor-initiating cells (TICs), tumorigenesis cells, pluripotent cells or clonogenic cells, are considered the origin of cancer cells. For their own origin, one hypothesis postulates that normal stem cells are a source to initiate carcinogenesis [10, 11], though a recent study shows that preexisting CSCs and normal stem cells are likely to differ in details [12]. Normal stem cells could transform into preexisting CSCs after cumulative genetic mutations, and this relationship exists theoretically and practically, as both cells can be isolated from normal and tumor tissues [13]. The CSCs of acute myeloid leukemia (AML) have a CD34^+^CD38^-^ cell surface marker resembling that of normal hematopoietic stem cells (HSCs) [14, 15]. Similarly in solid tumors, the CSCs of lung adenocarcinoma have the same Sca-1^+^CD34^+^ cell surface marker as bronchoalveolar stem cells (BASCs) [16]. Another hypothesis claims that differentiated somatic cells could be induced into CSCs with fetal phenotypes [17] and epigenetic abnormalities [18]. This hypothesis has been verified in distinct types of cancers. During the development of neoplasia in stomach, a fetal-like, dedifferentiated phenotype showed a low proliferation and beta-catenin accumulation, similar to stem cells [19]. Usually, the introduction of reprogramming factors (Oct-4, Sox-2, c-Myc, Klf4, etc.) are used in stemness transformation of somatic cells. *In vitro*-transformed fibroblasts can generate hierarchically organized tumors and obtain CSC properties, including the capability of self-renewal and differentiation along multiple lineages [20]. Non-tumorigenic mammary epithelial MCF-10A cells could induce the bulk of cells into tumorigenic CD44^+^CD24^low^ cells with CSC properties [21]. Sometimes, intervention measures including anticancer treatment play roles in the dedifferentiation. Combination of progesterone and irradiation could induce cancerous phenotype in MCF-10A cells and increase the proportion of radiation-resistant CSCs [22]. Also oncogenes show the critical function during the process of reprogramming. Glioma can originate from differentiated cells in the central nervous system (CNS) by transducing oncogenes into neural stem cells (NSCs), astrocytes, or even mature neurons in the brains of mice [23].

**Figure 1:**
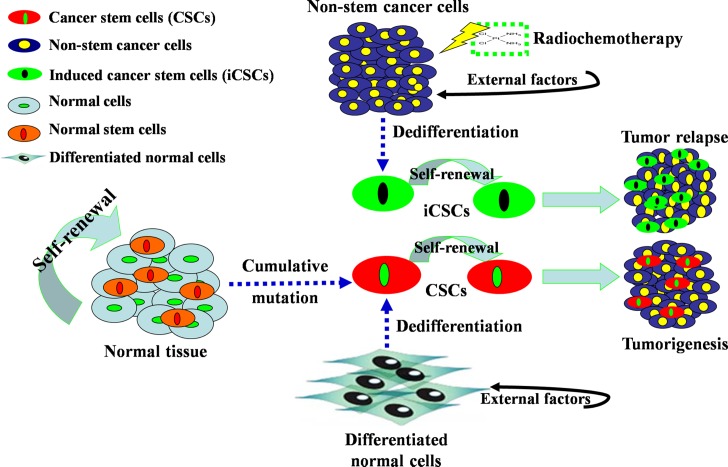
Origins of CSCs and iCSCs First, stem cells in normal tissues are forced to alter their differentiation pattern and become CSCs after long exposure to DNA-damaging agents that allows for cumulative mutations. Second, differentiated somatic cells can dedifferentiate to gain CSC properties with fetal phenotypes under the influence of external factors, such as Oct-4. Third, non-stem cancer cells can dedifferentiate into iCSCs under the influence of external factors and irradiation or chemotherapy. CSCs and iCSCs possess the capabilities of self-renewal, pluripotency and plasticity, and thus are considered the major cause of tumorigenesis and tumor recurrence.

Recent studies demonstrate that non-stem cancer cells can also be induced or dedifferentiate into CSCs and thus be a third origin of CSCs, and this subpopulation of CSCs is termed iCSCs [5]. Several methods including those mentioned above are reported in the procedure of this type of dedifferentiation. After down-regulation of mature neural genes and up-regulation of neural stem cell (NSC) genes, glioma tumor cells could dedifferentiate into iCSCs with the feature of drug resistance [24]. After retroviral introduction of stemness factors (Oct-3/4, Sox-2 and Klf4), human colon cancer cells displayed significantly enhanced CSC properties in terms of marker gene expression, sphere formation, chemoresistance and tumorigenicity [25]. When cultured under standard conditions *in vitro* or grafted subcutaneously into NMRI mice, human hepatocellular carcinoma (HCC) cells dedifferentiated into embryonic-development type (Hep3B) [26]. A similar study showed that CD138^high^ myeloma plasma cells possessed hematopoietic stem cell plasticity after being co-cultured with human osteoclasts for 20 weeks [27]. Low-temperature conditions also contribute to the dedifferentiation of non-stem glioma cells into stem-like cells [28]. In addition, external cytokines or transcription factors are other commonly used tools to convert non-stem cancer cells into iCSCs. For example, inhibitor of differentiation 4 (ID4) could dedifferentiate non-stem glioma cells into glioma stem cells [29]. IL-6 was sufficient to convert non-stem breast tumor cells into iCSCs [30], and Hiwi, a human homolog of the Piwi family, could promote tumorsphere formation of cervical cancer cells [31]. Moreover, epigenetic modifiers endowed neuroblastoma cells with stemness characteristics, which could be maintained for more than one year [32], and a potentially lethal damage also conferred cancer cells with stem-like features [33]. Interestingly, a report showed that non-stem breast cancer cells spontaneously dedifferentiated into stem-like cancer cells *in vitro* and *in vivo* [34]. All these findings indicate that “stem cell” and “dedifferentiation” theories can complement each other, and the dedifferentiation-redifferentiation process is critical for carcinogenesis (Figure 1).

## ICSCS DRIVEN BY RADIO-CHEMOTHERAPY

As established previously, preexisting CSCs are inherently resistant to ionizing radiation [35–38] and chemotherapy [39–42]. Preexisting CSCs in breast cancer, glioma, head and neck squamous cell carcinoma (HNSCC), colorectal cancer and pancreatic cancer have been found relatively resistant to radiochemotherapy compared to their non-tumorigenic progeny [36, 38, 43–45]. Other studies also report that ALDH^+^ and Side Population (SP) cancer cells are resistant to both ionizing radiation and chemotherapy [46, 47].

As the stem cell subpopulation enriches in the residual tumors after anticancer treatment, radiochemotherapy is generally used to enrich CSCs. Suspension culture combined with anticancer regimens is a strategy to screen breast cancer stem cells (BCSCs, CD44^+^CD24^-^) [48]. Recurrent HCC-derived cell lines were resistant to previously-used chemotherapy drugs, indicating features of CSCs [49]. Chemoresistant colorectal cells exposed to 5-Fu or oxaliplatin are enriched the CSC markers and displayed the CSC phenotype [45, 50]. In ovarian cancer cell lines, enrichment of CSCs was found in response to either cisplatin or paclitaxel or combination of both [51]. Similar results were reported in pancreatic cancer [44, 52], glioblastoma [53] and prostate cancer [54]. In our previous work, the combination of paclitaxel and serum-free medium cultivation enriched CSCs in lung adenocarcinoma A549 cells [55].

However, radiochemotherapy has recently been found to reprogram or dedifferentiate non-stem cancer cells into iCSCs [5]. Valproic acid (VA) is a histone deacetylase (HDAC) inhibitor regarded as a new class of anticancer agents able to protect normal cells and simultaneously sensitize cancer cells to ionizing radiation [6], and can promote the dedifferentiation of ALDH^-^ cells into ALDH^+^ cells and mammosphere-forming efficiency [7]. Differentiated breast cancer cells were reprogrammed into induced BCSCs (iBCSCs) after receiving ionizing radiation alone [8] or combination with progesterone treatment [22]. iBCSCs exhibited increased mammosphere-forming ability and tumorigenicity, and expressed the same genes related to stemness as BCSCs from non-irradiated samples [8, 22]. Not only in tumor cells as described above, but in breast cancer patients, a combination of genotoxic drugs (5-fluorouracil, doxorubicin, and cyclophosphamide) was reported to induce non-stem cancer cells into iCSCs. The therapy endowed non-stem cancer cells with stem-like features and induced multidrug resistance [56]. Furthermore, constitution analysis has also confirmed the existence of iCSCs. It is established that chromosomal aneuploidy is a hallmark of cancer cells and genome instability plays an important role in tumorigenesis, and CSCs are believed to be the initiator. To obtain direct evidence for the involvement of genomic instability in the induction of stem-like cancer cells (induced SLCCs, iSLCCs), Liang and coworkers [9] used multiple approaches (e.g. physical, chemical DNA damage inducers and UV lighting) to enhance genomic instability and found increased percentage of iSLCCs in cultured nasopharyngeal carcinoma cells (CNE-2) and neuroblastoma cells (SKN-SH). These findings suggest a novel origin of CSCs (i.e. iCSCs) responsible for cancer relapse after radiochemotherapy, which deserves further characterization.

In addition, despite knowledge of CSC enrichment during classic anticancer treatment [22, 44, 45, 48–55], it is unknown whether CSC expansion after radiochemotherapy derives from dedifferentiation of non-stem cancer cells, activation of dormant CSC phenotypes or increased cycling of preexisting CSCs. Nevertheless, it is certain that radiochemotherapy not only kills non-stem cancer cells, but also dedifferentiates non-stem cancer cells into iCSCs, a subpopulation resistant to radiochemotherapy. Collectively, concurrent investigations suggest that radiochemotherapy probably makes a triple contribution to the relative and absolute increase of CSCs. The first is a selective killing of non-tumorigenic cells. The second is a switch from an asymmetric type to a symmetric form of cell division that results in two proliferative daughter stem cells, giving rise to accelerated repopulation [8, 57]. The third is a dedifferentiation from non-stem cancer cells into iCSCs.

## MECHANISMS UNDERLYING GENERATION OF ICSCS BY RADIO-CHEMOTHERAPY

Preexisting CSC resistance to radiochemotherapy has been explained by a metabolic status associated with high levels of free radical scavenger [36, 58], low proteasome activity [59], activated DNA checkpoints [38], and expression of the ABC family including ABCG2, ABCB1, ABCB5 and other multi-drug resistance proteins [40, 60]. The mechanisms underlying iCSCs transformation via radiochemotherapy have not been fully understood. Recently, several signaling pathways, small non-coding RNAs (i.e. miRNAs) and tumor microenvironment have been reported to facilitate the dedifferentiation of cancer cells into cells with stemness phenotype.

### Signaling pathways of iCSCs

**Figure 2:**
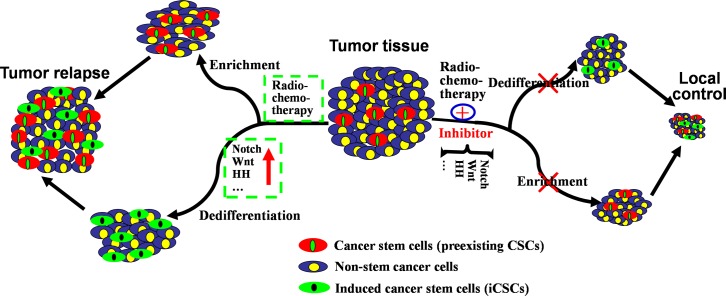
Mechanisms underlying the generation of iCSCs by radiochemotherapy and their implications for new therapeutic strategies Exposed to radiochemotherapy, preexisting CSCs in tumor tissues could survive and be enriched, and non-stem cancer cells could dedifferentiate into iCSCs via abnormally activated or upregulated signaling pathways (Notch, Wnt, HH, etc.). In this case, both preexisting CSCs and iCSCs exhibit increased mammosphere-forming ability, tumorigenicity and ability to cause cancer recurrence (left). Therefore, radiochemotherapy combined with pathways-inhibiting drugs could eradicate preexisting CSCs and prevent dedifferentiation of non-stem cancer cells into iCSCs. As a result, tumor tissue would dramatically shrink (right).

Three key pathways active in CSCs-related treatment resistance are Notch, Hedgehog and Wnt, in which alterations confer a limitless proliferative capacity to CSCs [37, 61]. These pathways also affect the dedifferentiation of non-stem cancer cells during radiochemotherapy. As CSCs are derived from normal stem cells [14–16], the pathways involved in normal stem cells, such as NF-κB, Notch, Wnt and Hedgehog, also function in CSCs [62]. Although transformation of normal stem cells into CSCs and dedifferentiation of non-stem cancer cells into iCSCs are two different processes, mechanistic differences have not been found. Theoretically, these pathways involved in transformation of normal stem cells into CSCs could accelerate repopulation and response of preexisting CSCs and iCSCs after radiation [57]. Under this condition, both preexisting CSCs and iCSCs exhibit increased mammosphere-forming capability, tumorigenicity and cancer recurrence (Figure 2, the left). In osteoblasts, radiation exerted effects on Notch signaling, as the expressions of Notch receptors (Notch1-4), ligands, target of Notch signaling (Hes1) and markers (ALP, M-CSF, RANKL and OPG) were altered following 2 and 4 Gy of irradiation [63]. In glioma, expressions of the constitutively active intracellular domains of Notch1 or Notch2 protect glioma stem cells against radiation [64]. In gastric cancer, prolonged Notch activation within dedifferentiated parietal cells finally enhanced cell proliferation and induced adenomas [65]. During the formation of iCSCs in the presence of irradiation, Notch signaling is coincided with up-regulation of reprogramming factors (Sox-2, Oct-4, Nanog, etc.), such as in iBCSCs [8]. Other studies revealed the role of Wnt signaling in the dedifferentiation of non-stem cancer cells. In a genetic model of intestinal epithelial cell (IEC), elevated NF-κB signaling enhanced Wnt activation and induced dedifferentiation of non-tumorigenic cancer cells [66]. In post-IR GBM cells, clonogenic cells with stem cell-like characteristics were enriched or induced and Wnt pathways were preferentially activated [67]. As for Hedgehog pathway in dedifferentiation of non-stem cancer cells, Hedgehog ligand was found highly expressed in GBM-derived neurospheres after irradiation, suggesting a potential mechanism of pathway activation required for the growth of preexisting CSCs and iCSCs during radiation response [68]. In addition, chemotherapeutic agents created an NFkB-IL6-dependent inflammatory environment to induce the stemness of non-stem cancer cells [56]. Generally, activated or upregulated signaling pathways take part in promoting the generation of iCSCs and the growth of preexisting CSCs. However, there remains no report on the difference between the signaling pathways in these two kinds of CSCs. Future research would focus on this field.

### Functional miRNAs in iCSCs

A group of miRNAs involved in the above signaling pathways promote stemness maintenance in normal stem cells and CSCs [55, 69]. MiR-125b has been shown as a key mediator of Snail-induced CSC enrichment and its elevated expression may be associated with chemoresistance [70]. Snail overexpression dramatically increased miR-125b expression and conferred chemoresistance to cancer cells through activated Wnt/β-catenin/TCF4 axis, which increased BCSC population (CD24^-^CD44^+^) [70]. In contrast, let-7 and miR-200 families have been shown to inhibit inappropriate inclinations to stemness and maintain differentiated cells in their differentiated state [71]. MiR-200c overexpression can increase radiosensitivity of human breast cancer cells by targeting TANK-binding kinase 1 (TBK1) [72]. Although these miRNAs are only reported in CSCs, they should be also involved in iCSCs. The concept of iCSCs was recently formulated, but they are possibly included in the so-called CSCs in previous studies. A direct proof from Cittelly at al. [73] demonstrates that suppression of miR-29 potentiates dedifferentiation of breast cancer cells into tumorigenesis state (actually iBCSCs) via Krüppel-like factor 4 (Klf4), a transcription factor required for the reprogramming of differentiated cells into pluripotent stem cells. All the above findings indicate that up- or down-regulation of miRNAs may assist in dedifferentiation of non-stem cancer cells.

### Microenvironment of iCSCs

Tumor microenvironment (tumor niche, heterogeneous stromal cells and oxygenation state in CSC origination and radiochemoresistance) is closely associated with stemness maintenance of CSCs [74, 75]. For example, Hyaluronan (HA), a major component of the extracellular matrix (ECM), is present in CSC niches and mediates activation of matrix metalloproteinase (MMP) signaling during tumor progression and CSC proliferation [76]. Tumor-associated macrophages (TAMs) promote CSCs-like phenotypes in murine breast cancer cells by up-regulating expressions of Sox-2, Oct-4, Nanog, ABCG2, and Sca-1, in addition to increasing resistance to chemotherapy and tumorigenicity *in vivo* [77]. Chemotherapy-induced hypoxia-inducible factors (HIFs) enrich BCSCs through IL-6 and IL-8 signaling and increase expression of multidrug resistance 1 [78]. Tumor microenvironment is also reported as a factor that converts non-stem cancer cells into iCSCs [79, 80]. Under the influence of different environmental niches, malignant cells derived from somatic mesenchymal stem cells (MSCs) could give rise to iCSCs with several stemness features including drug resistance [79]. Transforming growth factor β1 (TGF-β1) dramatically induces non-stem osteosarcoma cells into iCSCs with chemoresistance and metastatic potential [81]. Hypoxic environment also promotes tumor invasion and accelerates metastatic outgrowth of residual tumor cells in liver, and this fact is associated with dedifferentiation and increased colony-forming capacity of iCSCs [82]. For direct evidence of alterations in tumor microenvironment during radiochemotherapy and generation of iCSCs by radiochemotherapy, studies have demonstrated that radio- and chemotherapies induce the expression of several factors in various organs that create a prometastatic microenvironment [83, 84]. Specifically, radiochemotherapy can induce a pro-metastatic microenvironment contributing to treatment resistance for ovarian cancer cells in bone marrow and other organs (liver and lungs), and such cells show features of CSCs [85]. Actually, there should be a dynamic equilibrium of dedifferentiation-redifferentiation between non-stem cancer cells and iCSCs in response to microenvironmental variation [86]. The main mechanisms involved in formation of iCSCs during radiochemotherapy are listed in Table 1. The interactions among the factors in microenvironment, and the signaling pathways, functional miRNAs and other regulatory molecules deserve further study in future.

**Table 1:** Mechanisms underlying the generation of iCSCs by radiochemotherapy

Type of cancer	Anticancer treatment	Signaling pathway	Ref.
Breast cancer	Ionizing radiation	Notch	[22]
Lung adenocarcinoma	Chemotherapy	miR-29ab, miR-183, miR-17-5p, miR-127-3P	[57]
Osteoblast	Ionizing radiation	Notch1-4	[64, 65]
Stomach cancer	Chemotherapy	Notch, Wnt	[66]
Glioblastoma multiforme	Ionizing radiation	Wnt	[68]
Glioblastoma multiforme	Chemotherapy Ionizing radiation	Hedgehog	[69]
Breast cancer	Chemotherapy	miR-200c	[70]
Breast cancer	Chemotherapy	miR-125b, Wnt	[71]
Breast cancer	Ionizing radiation	miR-200c	[73]
Breast cancer	Chemotherapy	miR-29	[74]
Transformed tumors	Chemotherapy	Microenvironment	[79]

## IMPLICATIONS FOR NEW THERAPEUTIC STRATEGIES

As the presence of preexisting CSCs decreases therapeutic effects and predicts patient prognosis [16, 43] and radiochemotherapy induces non-stem cancer cells into iCSCs, a subpopulation of CSCs [87], it seems reasonable to propose that a cancer cure can be achieved only if preexisting CSC and iCSC populations are eliminated. A novel quantitative model involving dedifferentiation rate and drug-resistant mutation rate was designed to investigate the dynamics of tumor progression and to study the implications of a reversible iCSC phenotype for therapeutic interventions.

By understanding how preexisting CSCs escape from radiochemotherapy and how radiochemotherapy generate iCSCs, more efficient treatment will be developed to improve patient outcomes [88]. Since many pathways in normal stem cells also function in CSCs and iCSCs, including the notable examples of NF-κB, Hedgehog, Notch, and Wnt signaling, the drugs that target these pathways can be used in combination with radio- or chemotherapy to eliminate preexisting CSCs and to prevent the transformation of non-stem cancer cells into iCSCs.

In glioma, γ-secretase inhibitors (GSIs) that suppress Notch pathway enhanced the sensitivity of glioma stem cells to radiation at clinically relevant doses; knockdown of Notch1 or Notch2 sensitized glioma stem cells to radiation and impaired xenograft tumor formation [62, 64]. Hedgehog pathway blockade by cyclopamine prevented GBM cells from developing intracranial tumors in athymic mice [68]. HIF inhibitors overcame the resistance of BCSCs to paclitaxel or gemcitabine *in vitro* and *in vivo*, leading to tumor eradication [78]. Since chemotherapy associated NFkB-IL6-dependent inflammatory environment endowed non-stem cancer cells with stem-like features, aspirin which disrupted NFkB-IL6 feedback loop was combined with chemotherapy to prevent the induction of non-stem cancer cells into CSCs and to sensitize the tumor cells to chemotherapy, finally improving the recurrence-free survival of breast cancer patients [56]. All the above findings indicate the promising effects of pathways-targeting drugs on blocking the dedifferentiation of non-stem cancer cells into iCSCs.

Drugs or compounds that target other factors facilitating the growth of CSCs have also been demonstrated to be effective in eradicating preexisting CSCs and iCSCs. Anti-CD44s reduced the number of CSCs in cultured pancreatic cancer cells and xenograft tumors [89]. Salinomycin, a polyether ionophore antibiotic isolated from *Streptomyces albus*, has been shown to kill CSCs in different types of human cancers in preclinical trial of human xenograft mice and a few clinical pilot studies [90]. Photofrin II, among the most frequently used photosensitizers, sensitized a CSC-enriched U-87MG human glioblastoma cell to radiation and increased the percentage of apoptotic CSCs [91]. Afatinib, a small-molecule inhibitor of the tyrosine kinases of EGFR, HER2 and HER4, suppressed self-renewal capacity and tumorigenicity and eradicated CSCs by decreasing ABCG2 expression [92]. Caveolin-1 silencing sensitized chemotherapy of breast cancer by limiting the self-renewal ability and promoting the differentiation process of CSCs [93]. In the above studies, preexisting and induced CSCs were not distinguished, so both subpopulations may have been killed. Direct killing of iCSCs have been reported recently. Small molecules such as flavonoids and polycyclic polyprenylated acylphloroglucinols (PPAP) isolated from Cuban propolis showed considerable anti-iCSCs activity and promising therapeutic effects in neuroblastoma [94]. Inhibitors of Notch signaling (e.g. γ-secretase inhibitor) could partially suppress the function of reprogramming factors (Sox-2, Oct-4, Nanog, etc) involved in the formation of iBCSCs [8], and enhanced the radiation sensitivity of breast cancer cells [95]. Disulfiram (DSF), a drug that targets the NF-κB-stemness gene pathway, significantly inhibited generation of radiation-induced BCSCs in mice [8, 64, 96]. Additionally, trastuzumab emtansine (T-DM1) could target and deplete both preexisting and induced breast cancer stem cells [97]. Due to the possibly dynamic transformation of iCSCs into preexisting CSCs during the course of radiochemotherapy, different targeted sensitization strategies are needed at different stages of radiochemotherapy [5].

In conclusion, radiochemotherapy combined with drugs targeting the above signaling pathways and other factors that characterize CSCs could repress the growth of preexisting CSCs and generation of iCSCs. Thereafter, tumor tissue would dramatically shrink and be locally controlled (Figure 2, the right). Till now, little research shows differential killing between CSCs and iCSCs, which may be realized in the future. Reasonably, preexisting CSCs or quiescent cancer stem-like cells could be killed by inhibitors targeting the molecular abnormality in CSCs. For example, aberrant DNA hypermethylation is critical in regulating the renewal and maintaining the stemness of CSCs, and administration of decitabine (DAC), a DNA hypermethylation inhibitor [98], can overcome drug resistance caused by preexisting CSCs [89, 92]. However, the key point to eradicating iCSCs is to block the dedifferentiation of non-stem cancer cells into iCSCs and to prevent the molecular alterations in signaling pathways involved in the formation of iCSCs [8, 95, 96].

## PERSPECTIVE AND FUTURE

Recent progress in stem cell biology and reprogramming of somatic cells into a pluripotent phenotype has generated a new wave of excitement in regenerative medicine. Here we review the reports on the origin of CSCs, cancer cell reprogramming and mechanisms involved in iCSC formation, and propose some therapeutic implications. CSCs can be induced from non-stem cancer cells by radiochemotherapy, forming a subpopulation called iCSCs. This side effect of radiochemotherapy may become a novel cause of tumor recurrence and metastasis, arousing new interests in radiochemotherapy resistance. If iCSCs exist during radiochemotherapy, they will become new targets of cancer treatment. The therapeutic strategies targeting iCSCs must be able to block the dedifferentiation of non-stem cancer cells and to promote the terminal differentiation and tumorigenicity loss of iCSCs. Overall, any potential drugs facilitating the differentiation of CSCs or iCSCs would enhance the chemosensitivity of those preexisting CSCs or iCSCs as shown in a previous study [99]. However, the side effect from the potential differentiation therapeutics for normal stem cells should be warned as an alert. Even if proper reprogramming proves to be highly efficient for cancer cells, one should not expect miraculous cures from cell replacement therapies in the immediate future. Further study remains to be conducted. The role of the immune system and the potential of immunostimulatory agents may be considered to facilitate the treatment of cancer. Not only should blockage of preexisting CSCs and iCSCs be applied in cancer treatment, but recognition of these cells by the immune system is highly needed. This would be the ultimate goal of cancer treatment.

## Abbreviations

CSCs: cancer stem cells
iCSCs: induced cancer stem cells
AML: acute myeloid leukemia
TICs: tumor-initiating cells
CNS: central nervous system
NSCs: neural stem cells
HSCs: hematopoietic stem cells
EMP: epithelial mesenchymal plasticity
HNSCC: Head and Neck Squamous Cell Carcinoma
HDAC: histone deacetylase
BCSCs: breast cancer stem cells
iBCSCs: induced breast cancer stem cells
SLCCs: stem-like cancer cells
miRNAs: small non-coding RNAs
IEC: intestinal epithelial cell
ECM: extracellular matrix
TAMs: Tumor-associated macrophages.
